# Why should pharmacological trials in schizophrenia employ functional magnetic resonance imaging (fMRI)?

**DOI:** 10.1177/02698811211000773

**Published:** 2021-04-28

**Authors:** Evangelos Papanastasiou, Sukhwinder S Shergill

**Affiliations:** 1 Department of Psychosis Studies, Institute of Psychiatry, Psychology and Neuroscience, King’s College London, London, UK; 2 Therapeutic Area CNS, Retinopathies and Emerging Areas, Boehringer Ingelheim International GmbH, Ingelheim, Germany

To the Editor,

Drug discovery and development in CNS has been largely unsuccessful for several decades despite the pressure applied by significant unmet medical needs. This situation is typically seen in schizophrenia, where several compounds targeting negative or treatment-resistant positive symptoms did not succeed in meeting their primary clinical endpoints; more recent selected examples to include bitopertin (a glycine transporter 1 inhibitor developed by Roche as a treatment for the negative symptoms of schizophrenia), LuAF11167 (a phosphodiesterase 10A enzyme selective inhibitor developed by Lundbeck as a monotherapy for schizophrenia) and LuAF35700 (a dopamine, serotonin, noradrenaline receptor antagonist developed by Lundbeck for the management of treatment-resistant schizophrenia) and roluperidone (a 5-HT2A and σ_2_ receptor antagonist developed by Minerva as a monotherapy for schizophrenia). Given the elevated failure rates, a number of pharmaceutical companies have withdrawn their activities from schizophrenia, while declining support by the National Institute of Mental Health (NIMH, USA) has further aggravated the problem, thus limiting the treatment options available for patients and clinicians (Torrey et al., 2018).

There are two major problems in schizophrenia trials, both inherent in psychiatric research. First, the contemporary definition of schizophrenia broadly reflects the original clinical observations of Emile Kraepelin and Eugene Bleuler who coined the term in the late 19th and early 20th centuries. It was however recognised from an early age that this concept is a cluster of quite heterogeneous disorders, grouped together often arbitrarily (Kapur, 2011). Hopefully, in more recent years, research has focused on different groups of symptoms, that is, positive, negative and cognitive, which also received clinical attention in everyday practice. Second, the research practice is dominated by operationalised ‘psychometric’ scales as measures of clinical efficacy. The use of similar standardised tools has undeniably increased validity and reliability and enabled the introduction of clinical endpoints in assessing novel pharmacological compounds; however, the limited use of neuroscientific investigation did not allow to unveil the multiple effects these agents have on the brain, with the exception of positron emission tomography (PET) scans used to detect neuroreceptor binding.

Ongoing faith in an obsolete nosological classification system has obviously impeded the discovery of widely accepted surrogate biological markers for this group of disorders of intricate nature; this has inadvertently promoted overreliance on clinical measures as the only reliable means to investigate the effects of new compounds. There is scope however to reverse this picture and employ more brain biomarkers while investigating new drugs in schizophrenia, until we transition to a more dimensional psychiatric nosology, as advocated by the Research of Domain Criteria (RDoC) project of the NIMH (Insel, 2014). As the closest aspect to the mapping onto symptoms is at the brain circuit level, functional magnetic resonance imaging (fMRI) is the method of choice, allowing for analysis of functional connectivity within different brain regions; this can be further supplemented by electroencephalography (EEG) and magnetoencephalography (MEG) data to enhance the temporal resolution.

The traditional avenue to investigate the effects of new molecular entities into the brain has been at the neuroreceptor level; therefore, PET has emerged as the technology of choice for ‘molecular target engagement’. This approach however has limitations when one intends to capture the multiple effects of a new compound to more than one neuroreceptors, not to mention its inability to target those receptors for which labelling has not been developed yet. The use of fMRI could bridge this gap as ‘neurocircuit engagement’ normally reflects the summative activity of multiple neurotransmitter systems, hence more meaningful for the majority of compounds attacking several molecular targets.

Cognition has recently emerged as a novel pharmacological target in schizophrenia trial. Patients with schizophrenia suffer from various degrees of deficits in several cognitive domains, which usually present from an early age; impaired cognition together with negative symptoms are the major cause of the marked functional disability often associated with schizophrenia (Villalta-Gil et al., 2006). Besides, cognitive deficits have been increasingly recognised as primary biological processes across the whole psychosis continuum; those deficits could also account for the development of other symptoms seen in schizophrenia, as postulated by the aberrant salience model of psychosis (Kapur et al., 2005). Especially in the case of negative symptoms, efforts to differentiate or separate those from the underlying cognitive deficits often prove both misleading and artificial.

Cognition in schizophrenia is formally assessed via standardised batteries of cognitive tests, such as the Cambridge Neuropsychological Test Automated Battery (CANTAB) or the Measurement and Treatment Research to Improve Cognition in Schizophrenia (MATRICS) Consensus Cognitive Battery (MCCB). As demonstrated so far, fundamental behavioural cognitive measures such as reaction time and accuracy can be imperfect measures of cognition; a clear observation of neural activation is normally required in those circumstances where a divergence between behavioural and neural measures is expected, that is when the increased mental effort is needed to generate a certain level of cognitive performance (Wilkinson and Halligan, 2004). fMRI is a relatively cheap and safe method to investigate localised brain activation as a response to challenges induced by a variety of cognitive tasks; it might then present the optimum vehicle to investigate cognition in schizophrenia, by complementing the standard use of cognitive testing.

A particular difficulty envisaged during CNS and schizophrenia trials is the translation of preclinical findings to clinical results. Most candidate drugs fail to meet clinical endpoints, after having generated strong signals in behavioural preclinical studies. Though this observation lends support to the suspicion that animal models of psychosis poorly describe the human condition, it might be still possible to compare the efficacy of a new compound across species by revealing patterns of homologous brain circuits activation. fMRI can consequently offer a new avenue to investigate the ‘biological signature’ of a new compound by combining animal and human neuroimaging data; thus, preclinical studies could be optimised so as to provide a reliable evaluation of multiple pharmacological candidates and enhance due diligence prior to clinical testing (Borsook et al., 2006).

At later phases of clinical development, attrition rates can increase while progressing from phase I to phase III trials, for a variety of reasons, typically including overinflated placebo response. As suggested by the literature, fMRI can significantly reduce the cost of clinical development by either increasing the number of candidate molecules which enter the pathway (simple filter) or by informing decision making where multiple pathways are considered (Wise and Preston, 2010). A joint initiative from the International College of Psychopharmacology (CINP) and the Japanese Society of Neuropsychopharmacology (JSNP) has accepted the role of fMRI as a channel to investigate brain functional changes and has included neuroimaging data among CNS biomarkers needed to inform a safe transition from a phase I to a phase II/proof-of-concept (POC) trial (Suhara et al., 2017).

The potential advantages and clinical applications of fMRI in clinical trials of schizophrenia are summarised in Table 1. By supporting the use of fMRI, one should acknowledge the limitations of this method. As recently highlighted, variability in the analysis of neuroimaging datasets by different teams can have substantial effects on driven conclusions (Botvinik-Nezer et al., 2020); similar observations remind us of the need for extended sharing of experimental design and data analysis workflows within the scientific community, as well as reporting of multiple analyses outcomes of the same dataset. Besides, the dynamic nature of fMRI can account for a modest test–retest reliability of acquired imaging data. In contrast with PET imaging, which generates rather ‘absolute’ signals, fMRI is based on calculating differences in brain activation between different ‘states’ which typically correspond to different ‘phases’ of cognitive paradigms, targeting the neural circuits of interest. Therefore, the signal detected by fMRI depends not only on the selected activation task but also on the baseline state of the subjects; this can often impede the duplication of results. Increased awareness of these restrictions is necessary to ensure optimal use of fMRI and interpretation of its outcome.

**Table 1.** fMRI advantages and clinical benefits.

fMRI advantages	fMRI clinical benefits
Offers the best opportunity to model entire brain circuits – using analysis of functional connectivity within different regions of the brain – and assess causal influences through the use of dynamic causal modelling approaches	Safety of administration and low financial cost
Ability to investigate brain activation related to cognitive function and the resulting effect of a new drug	Can reduce the overall cost of clinical development
Ability to investigate aspects of brain function not detected by conventional cognitive testing, i.e. mental effort	Can increase the number of candidate compounds which enter clinical development (simple filter action)
By combining fMRI across species, homology between CNS circuits can be explored, and animal models can be validated	Can inform decision making when facing multiple clinical development options
A ‘biological signature’ of a particular pharmacological compound can be obtained by detecting a particular pattern of brain activation	Can increase confidence while moving between phase I and POC trials

Despite the gains of fMRI largely outweighing any additional costs, the pharmaceutical industry has so far demonstrated a strong resistance in adopting fMRI trials as an ordinary step of the clinical development pathway. The use of fMRI remains sporadic and limited and its greater benefits are widely questioned by applying disproportionate levels of scrutiny in search of ‘specific’ usefulness. A change in the minds of clinical scientists and physicians involved in the design and conduction of clinical development programmes is primarily needed, so to gradually acknowledge brain biomarkers as equally important to clinical measures; in the same tone, increased acceptability of these biomarkers by regulatory agencies such as the Food and Drug Administration (FDA) and European Medicines Agency (EMA) will drive the necessary change in the pharmaceutical development, but for fMRI to become a regular component of clinical trials, companies will also have to invest substantial resources and abandon their current conservative line.
